# Adherence to Human Colon Cells by Multidrug Resistant *Enterobacterales* Strains Isolated From Solid Organ Transplant Recipients With a Focus on *Citrobacter freundii*

**DOI:** 10.3389/fcimb.2020.00447

**Published:** 2020-09-16

**Authors:** José Ramos-Vivas, Itziar Chapartegui-González, Marta Fernández-Martínez, Claudia González-Rico, John Barrett, Jesús Fortún, Rosa Escudero, Francesc Marco, Laura Linares, Javier Nieto, Maitane Aranzamendi, Patricia Muñoz, Maricela Valerio, Jose María Aguado, Fernando Chaves, Irene Gracia-Ahufinger, Aurora Paez-Vega, Luis Martínez-Martínez, María Carmen Fariñas

**Affiliations:** ^1^Instituto de Investigación Valdecilla-IDIVAL, Santander, Spain; ^2^Service of Microbiology, Hospital Universitario Marqués de Valdecilla, Santander, Spain; ^3^Service of Infectious Diseases, Hospital Universitario Marqués de Valdecilla, Santander, Spain; ^4^New York University School of Medicine, New York, NY, United States; ^5^Infectious Diseases Department, Hospital Universitario Ramón y Cajal, Madrid, Spain; ^6^Service of Microbiology, Hospital Clínic-IDIBAPS, Universidad de Barcelona, Barcelona, Spain; ^7^Infectious Diseases Service, Hospital Clínic-IDIBAPS, Universidad de Barcelona, Barcelona, Spain; ^8^Infectious Diseases Unit, Hospital Universitario de Cruces, Barakaldo, Spain; ^9^Service of Microbiology, Hospital Universitario de Cruces, Barakaldo, Spain; ^10^Clinical Microbiology and Infectious Diseases, Hospital General Universitario Gregorio Marañón, Madrid, Spain; ^11^Infectious Diseases Unit, Hospital Universitario 12 de Octubre, Madrid, Spain; ^12^Service of Microbiology, Hospital Universitario 12 de Octubre, Madrid, Spain; ^13^Microbiology Unit, Hospital Universitario Reina Sofía, Córdoba, Spain; ^14^Maimonides Biomedical Research Institute of Córdoba (IMIBIC), Córdoba, Spain; ^15^Department of Microbiology, Universidad de Córdoba, Córdoba, Spain; ^16^Infectious Diseases Unit, Hospital Universitario Reina Sofía, Córdoba, Spain

**Keywords:** *Enterobacterales*, *Citrobacter*, epithelial cells, virulence factors, bacterial adherence, transplant recipient

## Abstract

Enterobacteria species are common causes of hospital-acquired infections, which are associated with high morbidity and mortality rates. Immunocompromised patients such as solid organ transplant (SOT) recipients are especially at risk because they are frequently exposed to antibiotics in the course of their treatments. In this work, we used a collection of 106 *Escherichia coli*, 78 *Klebsiella pneumoniae*, 25 *Enterobacter* spp., and 24 *Citrobacter* spp. multidrug resistant strains isolated from transplant patients (hepatic, renal or renal/pancreatic) in order to examine their ability to adhere *in vitro* to HT-29 human colon cells, and to determine if some adhesive characteristics are associated with prevalence and persistence of these strains. A total of 33 *E. coli* (31%), 21 *K. pneumoniae* (27%), 7 *Enterobacter* spp. (28%), and 5 *Citrobacter* spp. (21%), adhered to the colon epithelial cells. Two main adherence patterns were observed in the four species analyzed, diffuse adherence, and aggregative adherence. Under transmission electronic microscopy (TEM), most bacteria lacked visible fimbria on their surface, despite their strong adherence to epithelial cells. None of the strains studied was able to induce any cytotoxic effect on HT-29 cells although some of them strongly colonizing both cells and glass coverslips at high density. Some of the strains failed to adhere to the epithelial cells but adhered strongly to the cover-slide, which shows that microscopy studies are mandatory to elucidate the adherence of bacteria to epithelial cells *in vitro*, and that quantitative assays using colony forming unit (CFUs) counting need to be supplemented with pictures to determine definitively if a bacterial strain adheres or not to animal cells *in vitro*. We report here, for the first time, the aggregative adherence pattern of two multidrug resistant (MDR) *Citrobacter freundii* strains isolated from human patients; importantly, biofilm formation in *Citrobacter* is totally dependent on the temperature; strong biofilms were formed at room temperature (RT) but not at 37°C, which can play an important role in the colonization of hospital surfaces. In conclusion, our results show that there is a great variety of adhesion phenotypes in multidrug-resistant strains that colonize transplanted patients.

## Introduction

The *Enterobacterales* family is a heterogeneous group of Gram-negative bacteria, colonizing the intestinal tract of humans and animals. These bacteria are opportunistic pathogens, commonly isolated from nosocomial lung, urinary tract, intraabdominal, and bloodstream infections, and many of them in intensive care units (ICUs) (Cervera et al., 2012; Hollyer and Ison, 2018).

The precise mechanism by which these bacteria pass from the intestinal tract of patients to the environment to cause hospital outbreaks is poorly known, although several factors such as adherence to human intestinal cells and the formation of biofilms could be key in this process.

Colonization of the epithelium is the first step in enterobacterial pathogenesis and this ability has been demonstrated using different cell lines *in vitro*. For diarrhoeagenic *E. coli*, the ability to adhere to human epithelial cells is expressed as specific patterns. When these bacteria adhere to the cells as tight clusters, the adherence is called aggregative adherence (AA), and bacteria form aggregates between the cells and the surface (usually coverslips). When these bacteria adhere diffusely to the cell surface, the pattern is called diffuse adherence; and when bacteria adhere in small clusters to the cells the adherence pattern is called localized. The AA phenotype is well-known in enteroaggregative *E. coli* (EAEC) (Nataro et al., 1992), but was also demonstrated in *K. pneumoniae* (Favre-Bonte et al., 1995; Livrelli et al., 1996), and non-human strains of *Citrobacter freundii* (Bai et al., 2012). *Citrobacter* spp. and *Enterobacter* spp. are increasingly being recognized as causative agents of nosocomial infections and are an important reservoir of antimicrobial resistance determinants (Chavda et al., 2016; Majewski et al., 2017; Liu et al., 2018; Yang et al., 2018; Annavajhala et al., 2019). While *Klebsiella* and *E*. *coli* are versatile pathogens, less is known about the adhesion, virulence, and pathogenicity of *Enterobacter* and *Citrobacter* because these genera of bacteria are very heterogeneous. All of these bacteria are increasingly the cause of nosocomial infections in immunocompromised patients such as solid organ transplant (SOT) recipients (Kumar and Ison, 2019). These patients are especially at risk of developing infections by multidrug resistant (MDR) bacteria, since among other risk factors, they are frequently exposed to antibiotics in healthcare settings.

Knowing the adherence properties of these species will help us to understand bacterial persistence and dissemination from medical devices and hospital settings, and to improve decolonization protocols in patients colonized with these pathogens.

In this work, we collected strains of *K. pneumoniae, E. coli, Enterobacter* spp., and *Citrobacter* spp. from different SOT patients in order to examine their ability to adhere *in vitro* to HT-29 human colon cells, and to discuss if some adhesive characteristics could associated with prevalence and persistence of these strains. As we report here the aggregative adherence pattern of two MDR *Citrobacter freundii* strains isolated from human patients, we have further investigated their adherence characteristics by performing biofilm formation assays.

## Materials and Methods

### Human Population and Setting

The human prospective cohort study was conducted between August 2014 and April 2018 in seven University Hospitals from five Spanish regions. This national project (the ENTHERE Study) focused on the study of intestinal colonization and infections by multidrug resistant *Enterobacterales* (MDRE) in patients with kidney, liver and kidney/pancreas transplants (Ramos-Vivas et al., 2019).

### Ethical Approval and Informed Consent

The study was performed in accordance with the Declaration of Helsinki. The protocol was reviewed and approved by the Institutional Ethics Committee at all participating hospitals. The participating hospitals were: Hospital Universitario Marqués de Valdecilla (Santander), Coordinating Center; Hospital de Cruces (Bilbao); Hospital Clinic Universitari (Barcelona); Hospital Gregorio Marañón (Madrid); Hospital Universitario 12 de Octubre (Madrid); Hospital Reina Sofía (Córdoba); and Hospital Universitario Ramón y Cajal (Madrid). Informed consent was obtained from each patient, according to local standards.

### Bacterial Strains

Over a period of 33 months, from October 2014 to June 2017, a total of 243 MDRE isolates defined as AmpC-hyperproducers and/or extended-spectrum β-lactamases (ESBLs) or carbapenemase producers, were included in this study. MDRE were obtained from 108 patients with kidney or liver transplant or both kidney and pancreatic transplant from the ENTHERE Study. Among these, 106 were *E. coli*, 78 were *K. pneumoniae*, 25 were *Enterobacter* spp., 23 were *C. freundii* and 1 was *C. braakii*. As described previously (Ramos-Vivas et al., 2019), bacteria were isolated from rectal swabs (in the 48 h prior to transplant and weekly samples till 4–6 weeks after transplantation) and directly inoculated onto chromID® ESBL and chromID® CARBA chromogenic agar plates (bioMérieux, Marcy L'Etoile, France). Colonies were subcultured on blood agar and MacConkey agar and preliminary identification and antimicrobial susceptibility was performed using the Vitek 2 system (bioMérieux, France). Identification was confirmed by MALDI-TOF using the Vitek MS (bioMérieux) system, in accordance with the manufacturer's instructions. Stock cultures were frozen at −80°C with 20% (vol/vol) glycerol.

As a control for specific adherence experiments, one strain of *Corynebacterium striatum* was used. As a control for bacterial cytotoxicity, *Serratia liquefaciens* strain HUMV-3250 was used (Remuzgo-Martinez et al., 2013).

### Immunofluorescence Assays

HT-29 (ATCC® HTB-38™) human colon cells were cultured in McCoy's 5a medium (Gibco) and placed in 24-well tissue culture plates containing round glass coverslips. All strains were cultured overnight in 10 ml of Luria broth at 37°C with moderate shaking (175 rpm). Bacterial suspensions were washed in phosphate-buffered saline (PBS) and adjusted to approximately 5 × 10^9^ CFU ml^−1^. HT-29 cells were infected with bacteria at a multiplicity of infection (MOI, bacterium:eukaryotic cell ratio) of ~100:1. The infected plates were centrifuged for 4 min at 200 × g prior to incubation to promote adherence of bacteria to cells and to synchronize infections. Infected monolayers were then incubated at 37°C with 5% CO_2_ for 180 min. For *Citrobacter* strains, experiments were also conducted with McCoy's 5a medium containing 1% mannose. After infection, cells were washed four times and fixed with cold paraformaldehyde (3.2% in PBS) for 20 min at room temperature (RT). Then, cells were permeabilized with Triton X-100 (0.1% in PBS) for 5 min at room temperature and washed five times with PBS. Atto-488 phalloidin (Sigma), which binds polymerized F-actin, was used to identify actin filaments and fibers. After cytoskeleton staining, coverslips were mounted on glass slides with Fluoroshield mounting medium containing DAPI (Sigma Aldrich) to stain double-stranded DNA. All preparations were examined with a Nikon A1R confocal scanning laser microscope equipped with 403 nm and 488 nm lasers and with a differential interference contrast (DIC) filter. Images were captured at random with a ×40 Plan-Fluor 1.3 NA (Numerical Aperture), ×60 Plan-Apo 1.4 NA, or ×100 Apo-TIRF 1.49 NA objectives, and processed using the NIS-Elements 3.2 software. All immunofluorescence experiments for each strain were repeated at least 2 times and photographed by two different observers.

### Transmission Electron Microscopy (TEM)

Selected strains (two adherent and two non-adherent) for each species were examined by TEM after growth at 37°C in Luria broth. Bacteria were applied to Formvar-coated grids and were air dried. The cells were then negatively stained with 1% phosphotungstic acid in distilled water for 5 s and were examined with a JEM-1011 transmission electron microscope (JEOL) operating at 80 kV and equipped with an Orius SC1000 charge-coupled device (CCD) camera (Gatan).

### Biofilm Formation

Biofilm formation of *Citrobacter* strains was estimated in 24-well polystyrene plates (Corning, Costar) as previously described (Remuzgo-Martinez et al., 2015; Ramos-Vivas et al., 2019). Briefly, plates were incubated for 48 h at 37°C and at 25°C, in static. Planktonic cells were removed and wells containing biofilms were rinsed three times with distilled water and the remaining adherent bacteria were stained with 2 ml/well of crystal violet (CV). CV was extracted by acetic acid (33%) and the amount of dye was determined at 620 nm using a microplate reader (Multiskan FC; Thermo Fisher). In each experiment, results were corrected for background staining by subtracting the value for crystal violet bound to uninoculated controls. The assays were performed 4 times for each isolate and the mean ± SD was reported. *Citrobacter* strains were classified as non-adherent, moderate or strong biofilm producers using the following criteria: OD ≤ 0.05, non-biofilm producer; OD > 0.05–0.1, weak biofilm producer; OD > 0.1 strong biofilm producer.

### Confocal Laser Scanning Microscopy (CLSM)

Biofilm architecture of selected *Citrobacter* strains was studied in 4-well μ-slides (Ibidi, Martinsried, Germany) as previously described (Remuzgo-Martinez et al., 2015). Briefly, the slides were placed inclined (~45°) into an incubator to form a liquid-air interface and after 48 h at 37 or 25°C, unfixed planktonic cells were removed by rinsing with saline (0.85% NaCl), and bacterial viability within biofilms was determined by using the *Bac*Light LIVE/DEAD bacterial viability kit (Molecular Probes Inc.). A series of optical sections was obtained with a Nikon A1R confocal microscope; the excitation wavelengths were 488 nm (green) and 561 nm (red), and 500–550 nm and 570–620 nm emission filters were used, respectively. Images at the liquid-air interface were captured at random with a 20 × Plan Apo (NA, 0.75) objective. Reconstructions of confocal sections and quantitative measurements were performed using NIS-Elements software, version 3.2.

### Molecular Characterization of Resistance Genes

Standard PCR was used to amplify several genes encoding extended-spectrum β-lactamases (*bla*_TEM_, *bla*_SHV_, and *bla*_CTXM_) and carbapenemases (*bla*_KPC_, *bla*_VIM_, *bla*_IMP_, *bla*_NDM_, and *bla*_OXA−48_) as previously described (Ramos-Vivas et al., 2019). PCR multiplex plasmid-mediated AmpC (*bla*_CIT_, *bla*_FOX_, *bla*_MOX_, *bla*_DHA_, *bla*_ACC_, and *bla*_EBC_) was performed as described elsewhere (Perez-Perez and Hanson, 2002).

### Statistics

All data from biofilm assays were derived from four independent experiments. Statistical analysis of the data was carried out with Student's paired two-tailed *t*-test. *P* < 0.05 was considered statistically significant.

## Results

### Adherence Patterns of *Enterobacterales* Strains to HT-29 Cells

**Figure 1:**
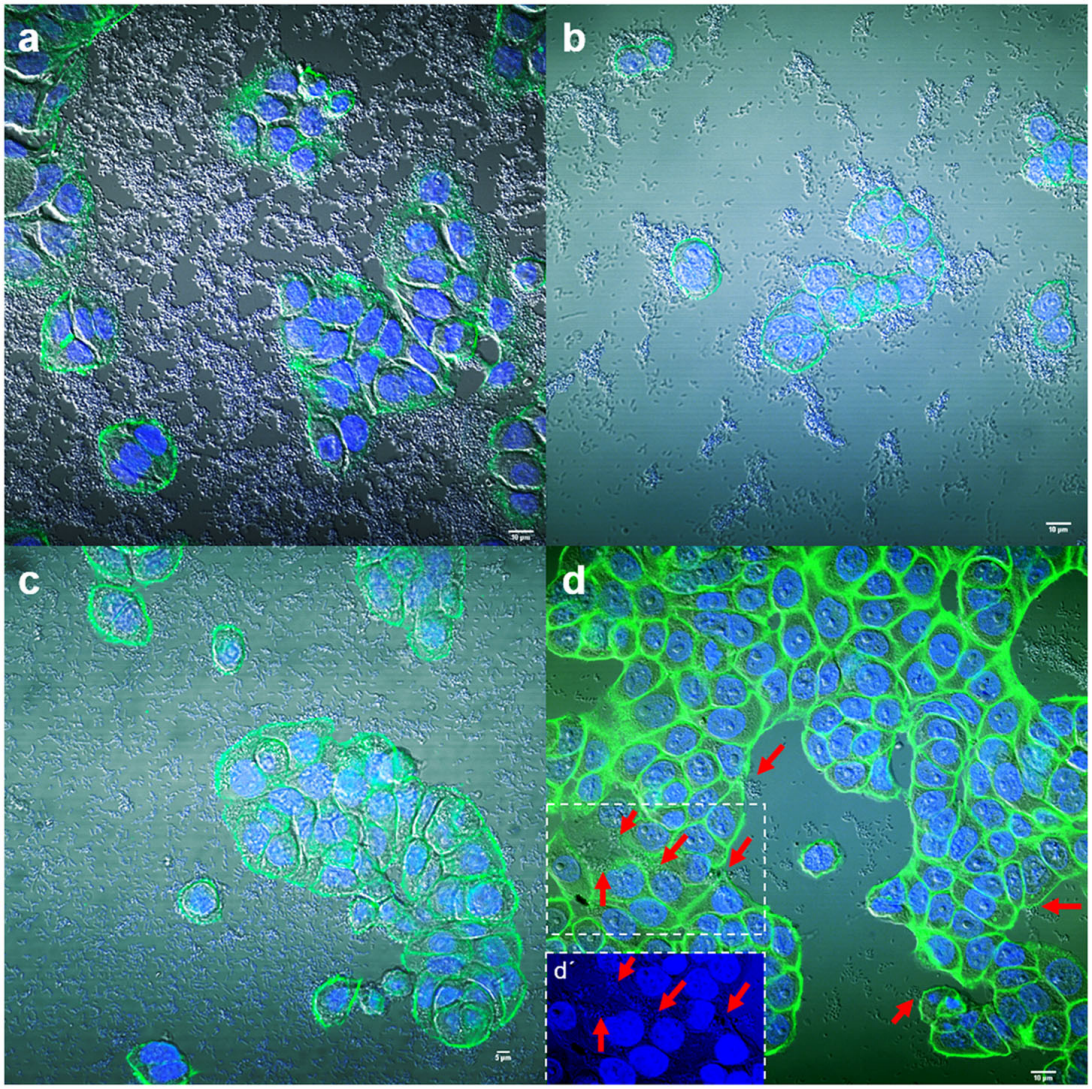
Representative images of aggregative adherence to HT-29 cells by *E. coli* strain 185 **(a)**, *Enterobacter* spp. strain 131 **(b)**, *Citrobacter freundii* strain 202 **(c)**, and *Klebsiella pneumoniae* strain 209 **(d)**. Arrows in **(d)** indicate some clusters of bacteria. **(d′)** Shows a detail of the DAPI channel from the boxed area in **(d)**. Cells were stained with Atto-488 phalloidin (green) and DAPI (blue). Scale bars: **(a,b,d)** 10 μm; **(c)** 5 μm. Original magnification: **(a,b,d)** ×600; **(c)** ×400.

**Figure 2:**
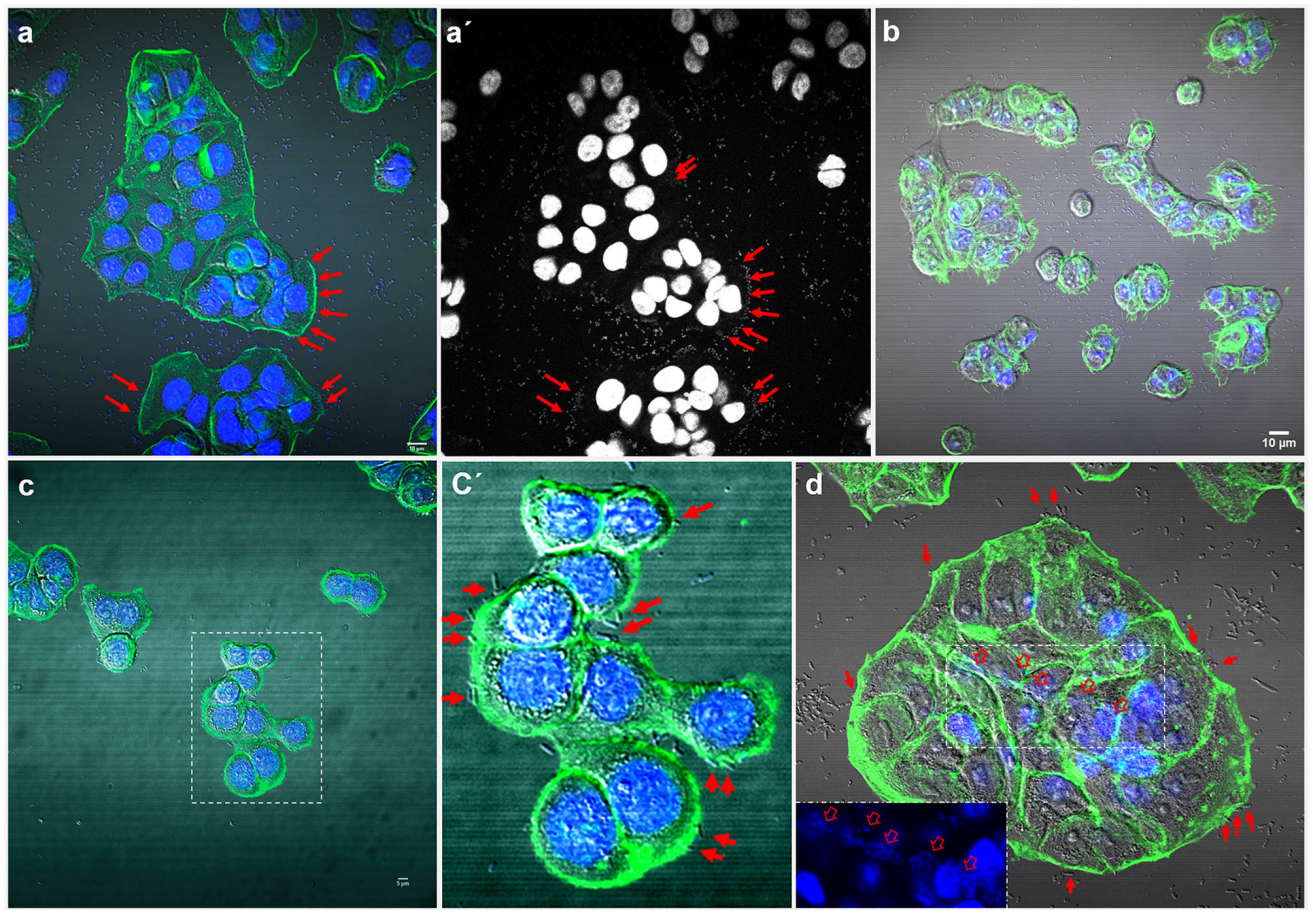
Representative images of diffuse and localized adherence to HT-29 cells by *E. coli* strain 186 **(a)**, *Enterobacter* spp. strain 136 **(b)**, *Citrobacter freundii* strain 152 **(c)**, and *Klebsiella pneumoniae* strain 195 **(d)**. **(a′)** shows the DAPI channel from **(a)**; where arrows indicate some attached bacteria. **(c′)** shows a detail of the boxed area in **(c)** and arrows indicate individual attached bacteria. In **(d)**, a central area of the HT-29 cells is shown with the DAPI channel in the boxed area. Arrows indicate individual attached bacteria at the edge of the cells and open arrows in the boxed areas indicate some clusters of bacteria on the cell surface. Cells were stained with Atto-488 phalloidin (green) and DAPI (blue). Scale bars: **(a,b)** 10 μm; **(c)** 5 μm. Original magnification: **(a,b,d)** ×600; **(c)** ×400.

Adhesion to human colon cells was considered positive if cells presented more than 1 bacteria per cell, after examining at least a quarter of the surface of two different coverslides and/or counting at least 100 eukaryotic cells. As shown in Table 1, a total of 33 *E. coli* (31%), 21 *K. pneumoniae* (27%), 7 *Enterobacter* spp. (28%), and 5 *Citrobacter* spp. (21%), adhered to the colon epithelial cells. Most strains, 73 *E. coli* (69%), 57 *K. pneumoniae* (73%), 19 *Citrobacter* spp. (79%), and 18 *Enterobacter* spp. (72%) did not adhere to this cell type (Supplementary Table 1). We observed two main patterns of adhesion, aggregative and diffuse, which are present in the four bacterial genera studied. Of these, 8 *E. coli* (7.5%), 4 *K. pneumoniae* (5%), 3 *Enterobacter* spp. (12%), and 2 *Citrobacter* spp. (8.3%) adhered to the colon epithelial cells in the so-called aggregative pattern, where large clusters of bacteria covered the cell surface or were located in large groups at the edge of the cellular cytoskeleton (Figure 1). DAPI staining helped us to demonstrate the presence of clusters of bacteria, since some strains do not differentiate well when they are on the cells (Figure 1d,d′). The other main pattern observed in most adherent strains was the diffuse adherence pattern, where the bacteria are randomly and individually attached to the edge of the cytoskeleton or over the cells (Figure 2).

**Table 1:** Strains with an adherent phenotype.

***K. pneumoniae***				***E. coli***			
**Strain**	N° weeks post-transplant^*^	**Transplant**	**Adherence**	**Strain**	N° weeks post-transplant^*^	**Transplant**	**Adherence**
25	1	Renal	Diffuse	5	3	Renal	Diffuse
30	2	Renal	Diffuse	6	1	Renal	Diffuse
40	4	Renal	Diffuse	7	6	Renal	Diffuse
69	1	Renal	Aggregative	22	3	Hepatic	Diffuse
83	0^**^	Renal	Diffuse	26	3	Renal	Diffuse
95	3	Hepatic	Aggregative	32	0^**^	Renal	Aggregative
110	0^**^	Renal	Aggregative	46	5	Hepatic	Aggregative
113	2	Renal	Diffuse	47	5	Renal	Diffuse
115	0^**^	Renal	Diffuse	51	4	Renal	Aggregative
116	1	Renal	Diffuse	57	1	Renal	Diffuse
135	3	Renal	Diffuse	68	4	Renal	Diffuse
138	3	Renal	Diffuse	79	0^**^	Renal	Diffuse
158	0^**^	Hepatic	Diffuse	85	5	Hepatic	Diffuse
159	2	Hepatic	Diffuse	93	0^**^	Hepatic	Diffuse
160	3	Hepatic	Diffuse	96	3	Renal	Aggregative
**163**	**Abdominal drainage**	**Hepatic**	**Diffuse**	99	0^**^	Hepatic	Diffuse
166	6	Renal	Diffuse	103	0^**^	Hepatic	Diffuse
178	2	Hepatic	Diffuse	104	0^**^	Hepatic	Diffuse
195	0^**^	Hepatic	Diffuse	114	2	Renal	Diffuse
201	2	Hepatic	Diffuse	124	2	Renal	Diffuse
209	2	Hepatic	Aggregative	126	3	Hepatic	Aggregative
				129	3	Renal	Aggregative
***Enterobacter cloacae***				137	0^**^	Renal	Diffuse
				149	0^**^	Renal	Aggregative
**Strain**	**N° week post-transplant**	**Transplant**	**Adherence**	150	2	Renal	diffuse
91	0^**^	Hepatic	Diffuse	154	1	Renal	Diffuse
120	1	Renal	Diffuse	181	3	Hepatic	Diffuse
**121**	**Blood**	**Renal**	**Diffuse**	184	6	Renal	Diffuse
131	3	Hepatic	Aggregative	185	2	Renal	Aggregative
136	1	Renal + pancreatic	Diffuse	186	1	Hepatic	Diffuse
155	2	Hepatic	Aggregative	198	0^**^	Renal	diffuse
156	3	Hepatic	Aggregative	**199**	**Urine**	**Renal**	**diffuse**
				**228**	**Urine**	**Renal**	**diffuse**
***C. freundii***							
**Strain**	**N° week post-transplant**	**Transplant**	**Adherence**				
144	1	Renal + pancreatic	Diffuse				
152	5	Hepatic	Diffuse				
177	0^**^	Hepatic	Diffuse				
202	6	Hepatic	Aggregative				
207	1	Hepatic	Aggregative				

*Strains that caused infection are highlighted in bold*.

* *Week in which the strain was isolated from rectal swab after transplantation*.

** *Strain isolated from rectal swab before transplantation*.

The so-called localized adherence pattern, was hard to see in our assays so, the adherence patterns in this work for all strains were divided into only diffuse or aggregative. The localized adherence was clearly demonstrated using a *Corynebacterium* strain as a positive control, which is able to adhere specifically and in a localized fashion to HT-29 cells (Supplementary Figure 1a).

**Figure 3:**
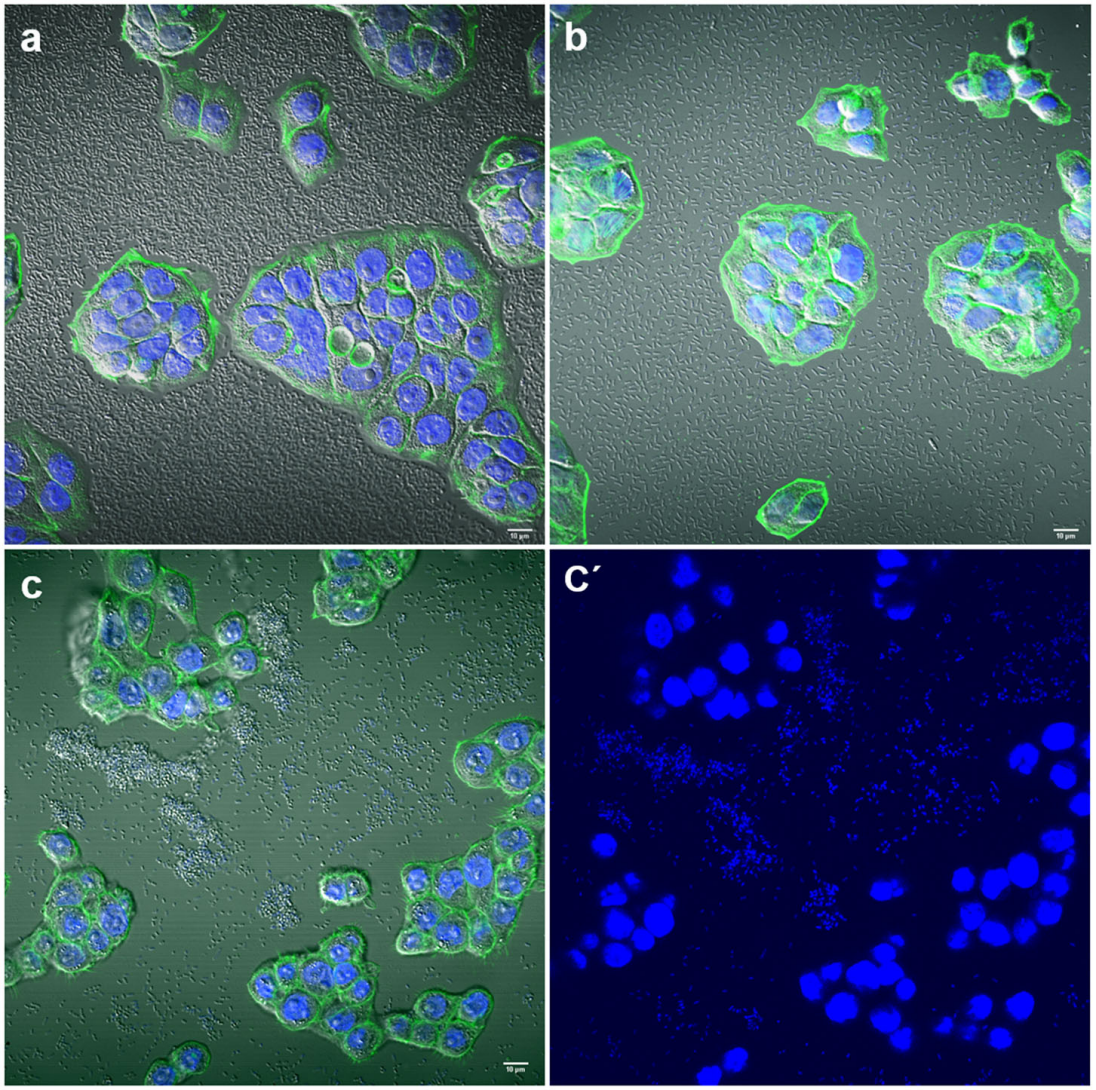
Representative images of negative adherence to HT-29 cells. **(a)** *E. coli* strain 143; **(b)** *Enterobacter* spp. strain 147; **(c,c′)** *K. pneumoniae* strain 25. Scale bars: **(a,b)** 10 μm; **(c)** 5 μm. Original magnification: **(a,b)** ×600; **(c,c′)** ×400.

Some strains adhered strongly to the glass coverslips. This adhesion to the inert surface seems to be very common for many isolates and should be taken into account or considered when carrying out quantitative studies on adherence to eukaryotic cells *in vitro* (Figure 3).

**Figure 4:**
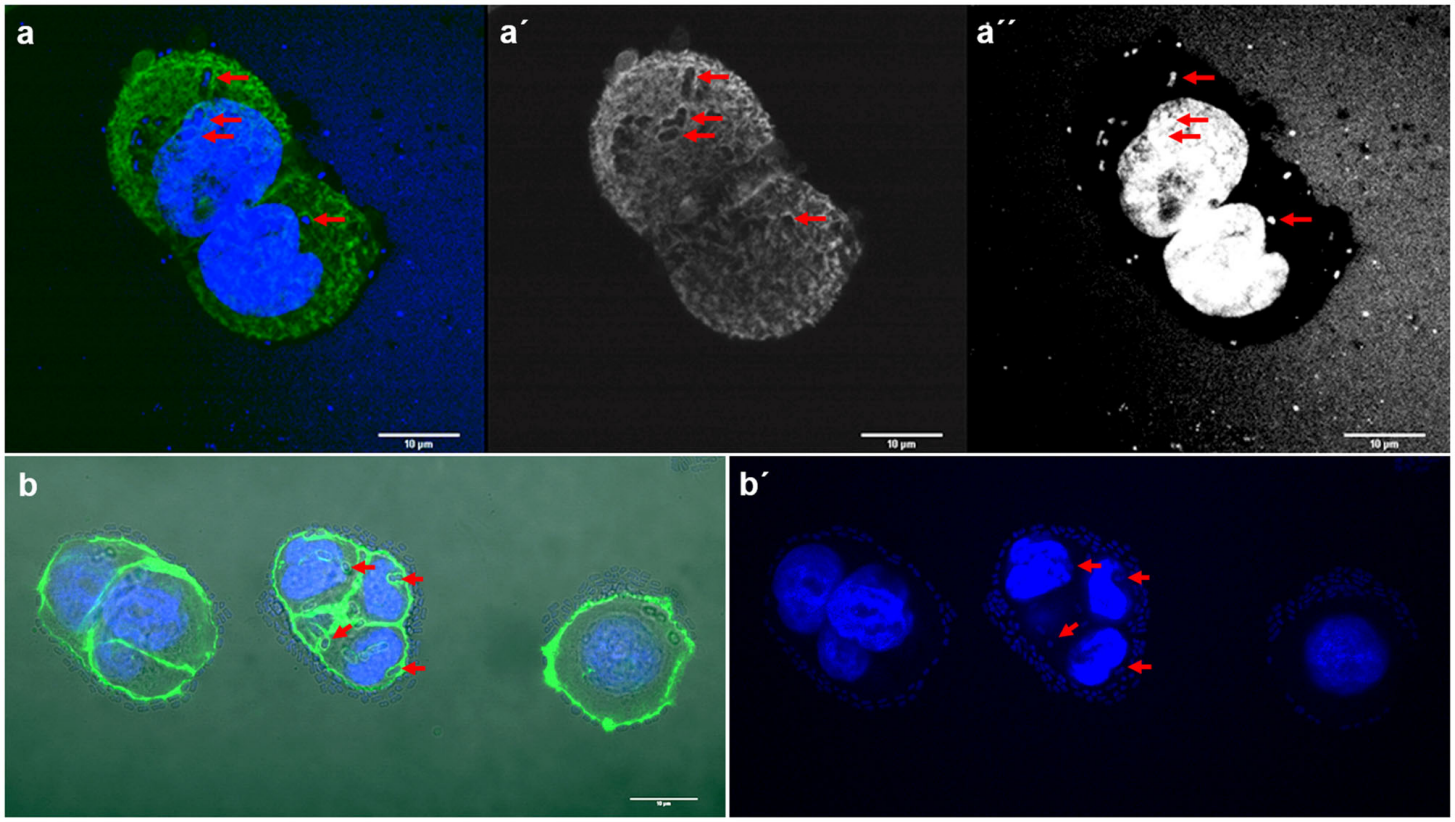
Representative images of *E. coli* and *K. pneumoniae* strains remodeling the actin cytoskeleton on the surface of HT-29 cells. Cells were stained with Atto-488 phalloidin (green) and DAPI (blue). Arrows indicate some bacteria forming pockets on the surface of HT-29 cells. **(a–a′′)** Maximum intensity projection of a 3D confocal image showing *E. coli* strain 154; **(b,b′)** *K. pneumoniae* strain 135. Scale bars: 10 μm; Original magnification: **(a–a′′)** ×600; **(b,b′)** ×400.

Interestingly, two strains, *E. coli* 154 and *K. pneumoniae* 135 appeared to form pockets on the surface of the cells, similar to actin pedestal formation (Figure 4). However, these bacteria are not intracellular, because they are not covered by the actin cytoskeleton. In fact, by confocal microscopy we have not detected any obvious signs that any of the strains were in an intracellular position. The study of the possible invasion or intracellular behavior of bacteria is beyond the objectives of this work.

HT-29 look healthy after 3 h post-infection in all strains as similar to uninfected cells used as control, with the cytoskeleton well-attached to the glass coverslide and the nuclei well-stained with DAPI, with no signs of cytotoxicity. As a positive control for cytotoxicity, *S. liquefaciens* strain HUMV-3250 was used; after 90 min of infection with this bacterium, the actin cytoskeleton staining revealed that the cells start to detach and the nuclei get smaller (Supplementary Figure 1b). Control for negative adherence (to cells or to coverslips), and control cells (no infection) are shown in Supplementary Figures 1c,d.

### Biofilm Formation by *Citrobacter* Strains

**Figure 5:**
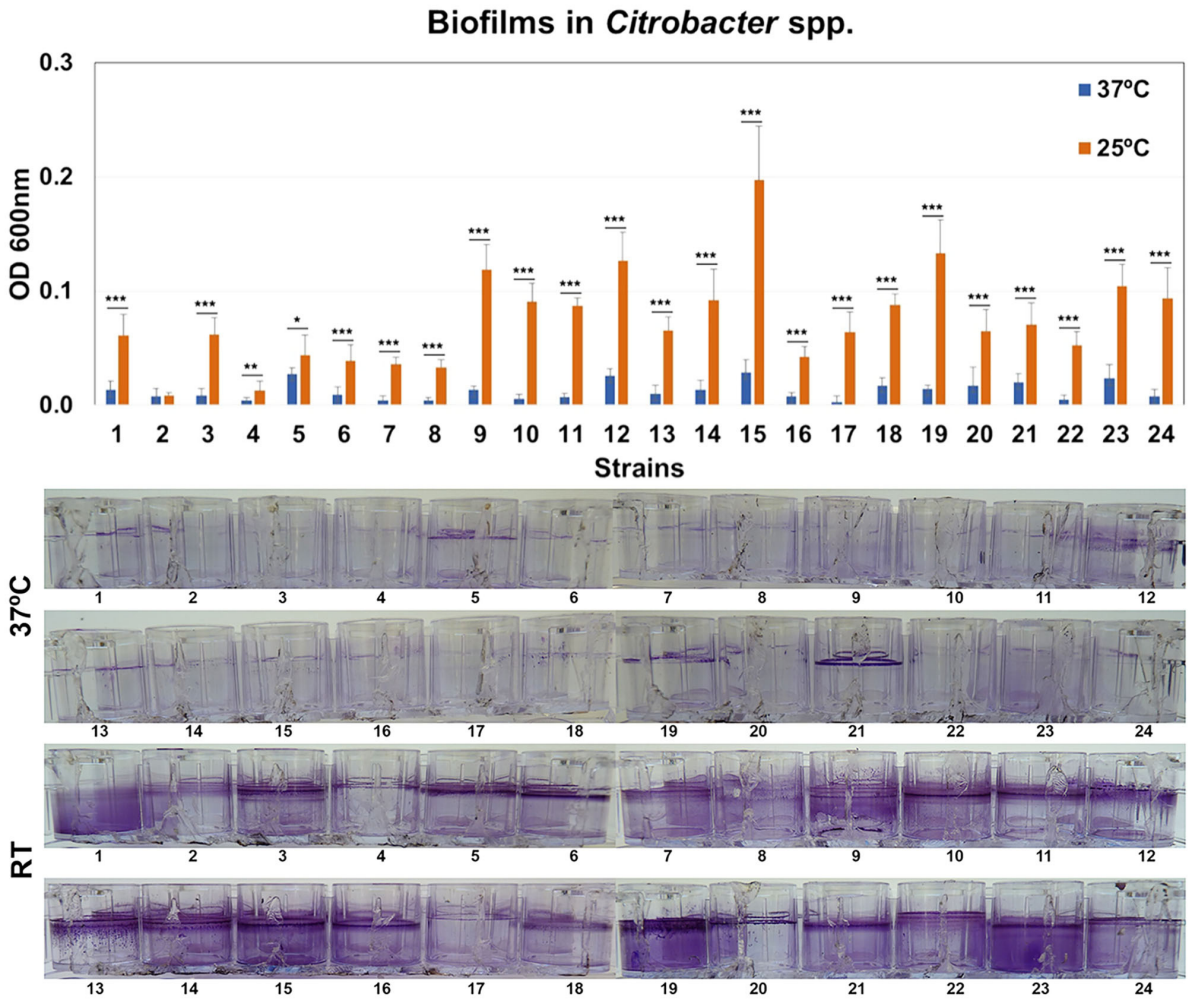
Quantification of biofilm formation. Biofilm formation by *Citrobacter* strains after growth at 37 or 25°C. Quantification of biofilm formation was performed after crystal violet extraction and measurement (OD_620_). Values are presented as the mean ± standard deviation (SD) of four independent experiments. *p < 0.05; **p < 0.01, and ***p < 0.001.

The ability of *Citrobacter* strains to form biofilms was quantified by crystal violet (CV) staining after 48 h at 37 or 25°C. All the biofilms were found at the liquid-air interface and were strongly influenced by temperature (Figure 5). Four representative isolates were selected for confocal microscopy analysis. Morphology of biofilms was found to be homogeneous but these strains showed weak or strong biofilm formation depending on temperature (Supplementary Figure 2).

### Surface Appendages

**Figure 6:**
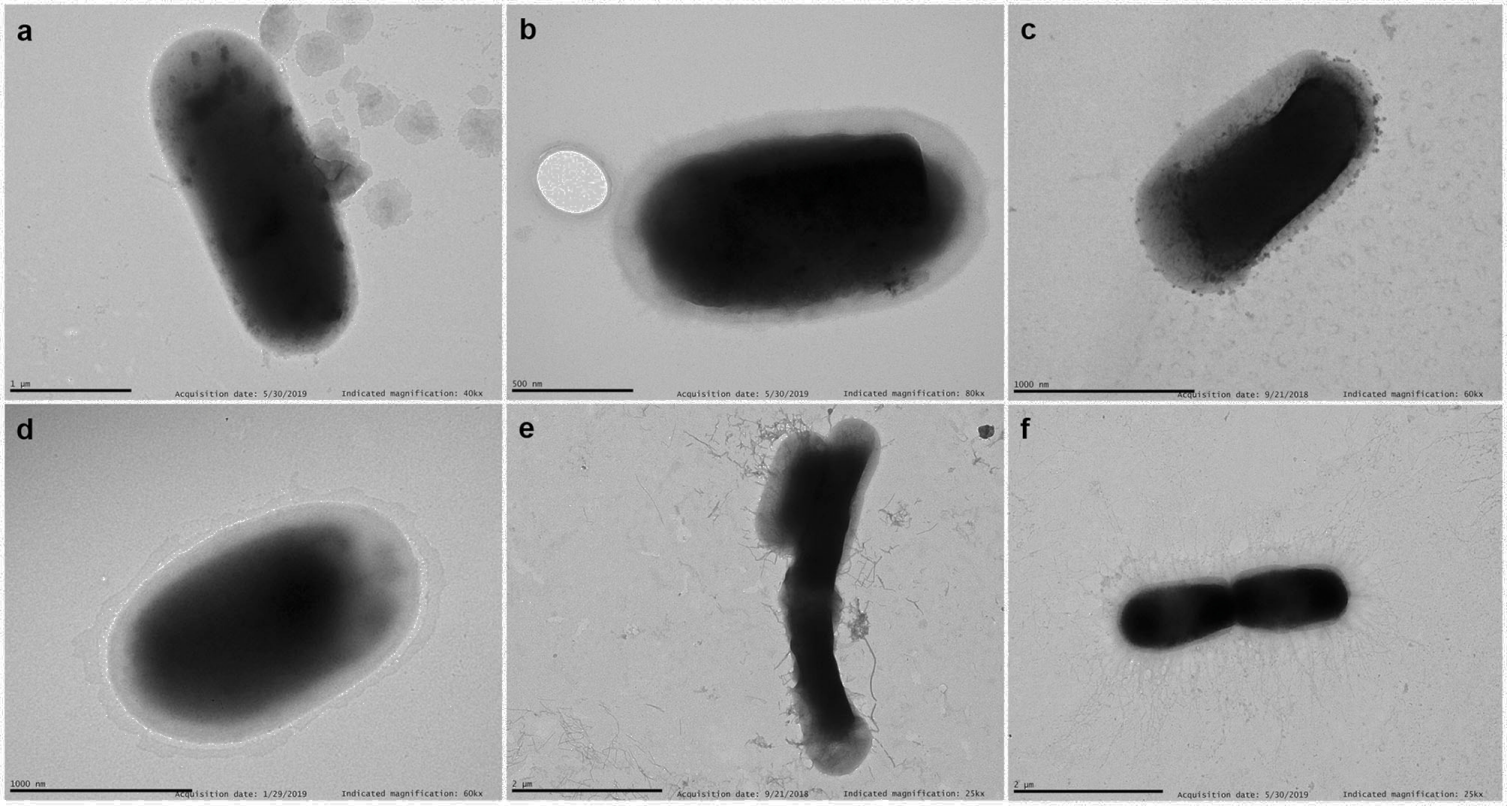
TEM. Examples of transmission electron micrographs of negatively stained bacteria grown overnight in Luria medium at 37°C. **(a)** *E. coli* 46 (aggregative adherence); **(b)** *Enterobacter* 155 (aggregative adherence); **(c)** *K. pneumoniae* 25 (diffuse adherence); **(d)** *C. freundii* 207 (aggregative adherence); **(e)** *Enterobacter* 121 (diffuse adherence); **(f)** *K. pneumoniae* 135 (diffuse adherence). Note the detachment of pili from the bacterial surface of *Enterobacter* 121. Scale bars indicate: **(a)** 1 μm; **(b)** 0.5 μm; **(c)** 1 μm; **(d)** 1 μm; **(e)** 2 μm; **(f)** 2 μm. Magnification: **(a)** ×40,000; **(b)** ×80,000; **(c,d)** ×60,000; **(e,f)** ×25,000.

Representative TEM images of selected strains are shown in Figure 6. We have chosen a diffuse adherent and a localized adherent strain of each species to study its surface by TEM. Strains of *E. coli*, and *Citrobacter* (diffuse or aggregative adherence) grown overnight on Luria broth showed a smooth surface. In contrast, piliated cells appeared clearly in diffuse adherent *Enterobacter* spp. and *K. pneumoniae* strains.

### Antibiotic Resistance in *Citrobacter* Strains

All *Citrobacter* isolates were clonally unrelated according to PFGE analysis (unpublished results). All strains tested except number 10 and 21 (CTXM-group 9), and strain 14 (CTXM-group 1) were positive for the overproduction of intrinsic chromosomal AmpC β-lactamases (Supplementary Table 1).

## Discussion

Since adherence of bacteria to cells is considered an essential step in bacterial pathogenesis, adherence to host cells *in vitro* provides a useful tool to investigate the interactions between pathogens and the human epithelium that occurs during infections.

Moreover, the study of colonization in SOT recipients by MDR enterobacteria may help to understand the role in infections caused by these colonizing microorganisms, considering that those patients are frequently exposed to antibiotics.

In this way, the HT-29 cell line is a very suitable model to study adherence by enteric pathogens, because these cells share morphological and functional features with normal colon cells (Gagnon et al., 2013). Therefore, the use of human colon cells can help us understand the colonization of the human intestine by these opportunistic pathogens. In this study, the adherence ability varied extensively among the strains used, and was independent of the type of transplant.

Phenotypic heterogeneity in adhesion was obvious in all species. We have not carried out quantitative studies of CFUs counts because most of the strains of the four species used in this study adhered poorly to HT-29 cells, but many of these adhered strongly to the glass surface. In the *in vitro* quantitative adherence experiments, CFU numbers were commonly used to classify bacteria as adherent or not adherent to human cells, assuming that all colony forming units obtained on bacteriological media after lysis of cell-infected cultures came from cell-attached bacteria. We note that many of these quantitative experiments lack microscopy. If microscopy is not used to check if the bacteria are really attached to the cells, it cannot really be said that the bacteria that are being used in these host-pathogen interaction studies were really adherent (Lazaro-Diez et al., 2016). Besides not using microscopy to observe if the bacteria are really attached to the cells or to the substrate, a positive control of adhesion is not commonly used; for example, with a bacterium that only adheres to the cells and not to the surface where these cells are growing. This means that microscopy photographs should be mandatory to evaluate the adherence of bacteria to cells, and that large microscope fields need to be showed to really verify bacterial adherence. Our positive control (*C. striatum*) demonstrated higher levels of specific adherence to these cells, and not to the substrate (glass) further validating our model.

We observed two different adhesion patterns, aggregative, and diffuse. The so-called localized adherence pattern, reported elsewhere for some strains of *E. coli, K. pneumoniae*, and *Enterobacter* spp. (Livrelli et al., 1996; Scaletsky et al., 1999; Mange et al., 2006) where small clusters are attached to the cells, were hard to see in our assays so, the adherence patterns in this work for all strains were divided only into diffuse or aggregative. Our results showing adherence heterogeneity are not new, because strains of the genera *Klebsiella, Escherichia*, and *Enterobacter* use different patterns to bind epithelial cells (Livrelli et al., 1996; Lopes et al., 2005; Mange et al., 2006; Krzyminska et al., 2010; Alcantar-Curiel et al., 2013; Kalita et al., 2014). However, there is a paucity of research using human colon cells to study the adherence of enterobacteria. Using colon cells is interesting because they can give us an idea of the potential of these strains to remain in the intestine of colonized patients. Only 17 strains (8 *E. coli*, 4 *K. pneumoniae*, 3 *Enterobacter cloacae*, and 2 *Citrobacter freundii*) adhered to the colon epithelial cells in the so-called aggregative pattern. The other main adherence pattern was observed in 49 strains (where bacteria are randomly attached to the edge of the cytoskeleton or over the cells).

Some strains of *K. pneumoniae* and *E. coli* appear to form pockets, similar to actin pedestal formation but generating structures toward the inside of the cell, in the inverse sense to the actin pedestals exhibited by Enterohemorhagic *E. coli* (EHEC) (Battle et al., 2014; Mcwilliams and Torres, 2014); these structures generated toward the inside of the eukaryotic cell merit further study, because *in silico* analysis of the so far sequenced *K. pneumoniae* genomes does not identify any T3SS components, although they are present in EHEC, EPEC, and *Citrobacter rodentium* (Kelly et al., 2006; Cano et al., 2009; Gaytan et al., 2016; Marcoleta et al., 2018).

Overall, the pattern of adherence is not related to the type of transplant. Also, when we have observed the relationship between adhesion and the presence of fimbriae on the surface of bacteria, we have not found any correlation. Some aggregative strains do not have visible fimbriae and others do, both in *Klebsiella* and *Enterobacter*.

We report for the first time the aggregative adherence pattern of two multidrug resistant (MDR) *C. freundii* strains isolated from human patients. Both strains produce a large biofilm when grown at 25°C. Other authors have found a strain of *C. freundii* isolated from goat that has a similar pattern of adherence, and that was also toxic to HEp-2 cells (Bai et al., 2012), indicating that this type of adherence may play a role in the pathogenicity of the bacterium. Adherence of the strains was not inhibited by the addition of 1% mannose. Furthermore, the aggregative strains did not show any visible fimbriae on their surface, as observed by transmission electron microscopy although, as in some *Enterobacterales*, the expression of fimbria could be regulated by temperature (Dorman and Ni Bhriain, 1992; Padilla et al., 2009; Hinthong et al., 2015) which could be involved in the formation of biofilms at room temperature but not at 37°C. Our *Citrobacter* strains were not cytotoxic, but both were multi-resistant, carrying a CTX-M extended-spectrum β-lactamase and hyperproduction of chromosomal AmpC, which should be taken into account when studying the biology of these pathogens in hospitals.

Cytotoxicity was reported in *Citrobacter* strains when HEp-2 cells (human larynx carcinoma) were used (Liu et al., 2018). Perhaps the difference in the results is that colon cells resist the presence of enterobacteria better, although the differences could also be explained by the different methods used to quantify cytotoxicity.

In this work, the percentage of strains of *Citrobacter* producing biofilm at 37°C was much lower than *E. coli* or *Klebsiella* strains and was similar to *Enterobacter* strains (Ramos-Vivas et al., 2019). In fact, the rate of biofilm formation by *Enterobacter* strains previously reported was very low (Ramos-Vivas et al., 2019). However, most *Citrobacter* strains produce strong biofilms after growth at room temperature, which can favor its persistence in the hospital environment. This finding could suggest that these strains can use a different strategy to persist in the host and to cause disease.

We also studied 6 strains that caused infections after transplantation. Of these, 3 showed a diffuse adherence pattern (*E. coli* 199, *E. cloacae* 121, and *K. pneumoniae* 163) and 3 were non-adherent (*K. pneumoniae* strains 27, 169, and 172). No strain with an aggregative pattern caused infection in transplant patients. Overall, these results indicate that adherence to colon cells by this large collection of strains is puzzling, but some strains that have caused infection and others that have an oriented phenotype to establish themselves in the cell's cytoskeleton deserve further investigation.

In this report, we have demonstrated that *Enterobacterales* strains display a high degree of phenotypic variability, which reinforces the relevance of monitoring the potential adverse impact of MDRE species in immunocompromised patients such as SOT recipients. Moreover, highly adherent strains can persist in the host in high numbers and can act as a reservoir of antimicrobial resistance genes so, the understanding of the interplay between those strains and the host could help us to design new strategies or treatments against them. Furthermore, these data, and recent concerning findings on MDR *Citrobacter* clinical isolates (Majewski et al., 2017; Venditti et al., 2017; Yang et al., 2018), call for further efforts to study the significance of *Citrobacter* in hospital settings.

## Data Availability Statement

All datasets presented in this study are included in the article/Supplementary Material.

## Ethics Statement

The studies involving human participants were reviewed and approved by the study was performed in accordance with the Declaration of Helsinki. The protocol was reviewed and approved by the Institutional Ethics Committee at all participating hospitals. The participating hospitals were: Hospital Universitario Marqués de Valdecilla (Santander), Coordinating Center; Hospital de Cruces (Bilbao); Hospital Clinic Universitari (Barcelona); Hospital Gregorio Marañón (Madrid); Hospital Universitario 12 de Octubre (Madrid); Hospital Reina Sofía (Córdoba); and Hospital Universitario Ramón y Cajal (Madrid). Informed consent was obtained from each patient, according to local standards. Informed consent was obtained from each patient. The patients/participants provided their written informed consent to participate in this study.

## Author Contributions

JR-V, IC-G, MF-M, CG-R, and MF conceived and designed the experiments and analyzed the data. JR-V, IC-G, MF-M, CG-R, and JB performed the experiments. JR-V and MF wrote the paper. All authors have contributed strains, read, and approved the final manuscript.

## Conflict of Interest

The authors declare that the research was conducted in the absence of any commercial or financial relationships that could be construed as a potential conflict of interest.

## Members of ENTHERE Study Group, the Group for Study of Infection in Transplantation of the Spanish Society of Infectious Diseases and Clinical Microbiology (GESITRA-SEIMC) and the Spanish Network for Research in Infectious Diseases (REIPI)

Carlos Armiñanzas^3^, Francisco Arnaiz de las Revillas^3^, Jorge Calvo^2^, Fernando Casafont-Morencos^15^, Emilio Fábrega^15^, Concepción Fariñas-Álvarez^16^, Mónica Gozalo^2^, Emilio Rodrigo^17^, Juan Carlos Ruiz San Millán^17^, Carlos Salas ^1,2^, Pilar Martin Dávila^5^, Patricia Ruiz Garbajosa^18^, Ana Sánchez-Díaz^18^, Adolfo Martínez^19^, Asunción Moreno^7^, Marta Bodro^7^, Maria Fernanda Solano^7^, María José Blanco^8^, Miguel Montejo^8^, Caroline Agnelli Bento^10^, Emilia Cercenado^10^, Mansilla, Marina Machado^10^, María Olmedo^10^, Cristina Rincón Sanz^10^, Teresa Vicente Rangel^10^, María Luisa Rodríguez Ferrero^20^, Sara Rodríguez Fernández^10^, María Luisa Rodríguez-Ferrero^20^, Luis Alberto Sánchez Cámara^20^, Elena Resino^11^, Fernando Rodríguez^13^, Julian Torre-Cisneros^14^. ^1,3^Infectious Diseases Service. Hospital Universitario Marqués de Valdecilla, Instituto de Investigación Valdecilla-IDIVAL, University of Cantabria. ^1,2^Service of Microbiology, Hospital Universitario Marqués de Valdecilla, Santander, Spain. ^5^Infectious Diseases Department, Hospital Universitario Ramón y Cajal, Madrid, Spain. ^7^Infectious Diseases Service, Hospital Clínic-IDIBAPS, Universidad de Barcelona, Barcelona, Spain. ^8^Infectiuos Diseases Unit, Hospital Universitario de Cruces, Barakaldo, Spain. ^10^Clinical Microbiology and Infectious Diseases, Hospital General Universitario Gregorio Marañon, Madrid, Spain. ^11^Infectious Diseases Unit, Hospital Universitario 12 de Octubre, Madrid, Spain.^13^ Microbiology Unit, Hospital Universitario Reina Sofía, Córdoba, Spain. Maimonides Biomedical Research Institute of Cordoba (IMIBIC), Córdoba, Spain. Department of Microbiology, Universidad de Córdoba, Córdoba, Spain. ^14^Infectious Diseases Unit, Hospital Universitario Reina Sofía, Córdoba, Spain. ^15^ Liver Unit, Hospital Universitario Marqués de Valdecilla Santander, Spain. ^16^Nephrology Department, Hospital Universitario Marqués de Valdecilla, Santander, Spain. ^17^ Quality Unit, Hospital Universitario Marqués de Valdecilla, Santander, Spain. ^18^Service of Microbiology, Hospital Universitario Ramón y Cajal, Madrid, Spain. ^19^Transplant Coordination, Hospital Universitario Ramón y Cajal, Madrid, Spain. ^20^Department of Nephrology, Hospital General Universitario Gregorio Marañon, Madrid, Spain.

## Supplementary Material

Supplementary Figure 1 *C. striatum* was used to determine specific adherence to HT-29 cells and as a control for localized adherence to HT-29 cells. Cells were infected for 3 h. *C. striatum* and the bacteria stained with a polyclonal antibody are shown in pink. The image in **(b)** was intentionally overexpose to show DAPI staining nuclei and bacteria. **(c)** Shows a non-adherent strain (neither to the cells nor the coverslips) and one control slide with cells alone **(d)**. Arrow indicates a single bacterium to account for the assay. Magnification **(a,c,d)** ×400; **(b)** ×600. Scale bars indicate: **(a,c,d)** 10 μm; **(b)** 5 μm.

Supplementary Figure 2 CLSM of biofilm formation by selected *Citrobacter* strains at different temperatures. Representative examples of CLSM images of selected strains after biofilm formation. Bacteria were stained with the *Bac*Light LIVE/DEAD viability kit. Live cells fluoresce in green with Syto 9 dye and dead cells are stained red with propidium iodide. Original magnification: ×200.

Supplementary Table 1 Strains that did not show adherence to colon cells. Strains that caused infection are highlighted in bold.
