# Supplementary figures and images for: Adherence to Human Colon Cells by Multidrug Resistant Enterobacterales Strains Isolated From Solid Organ Transplant Recipients With a Focus on Citrobacter freundii
Source: Front Cell Infect Microbiol. 2020 Sep 16;10:447. doi: 10.3389/fcimb.2020.00447 (PMC7525035; doi:10.3389/fcimb.2020.00447)

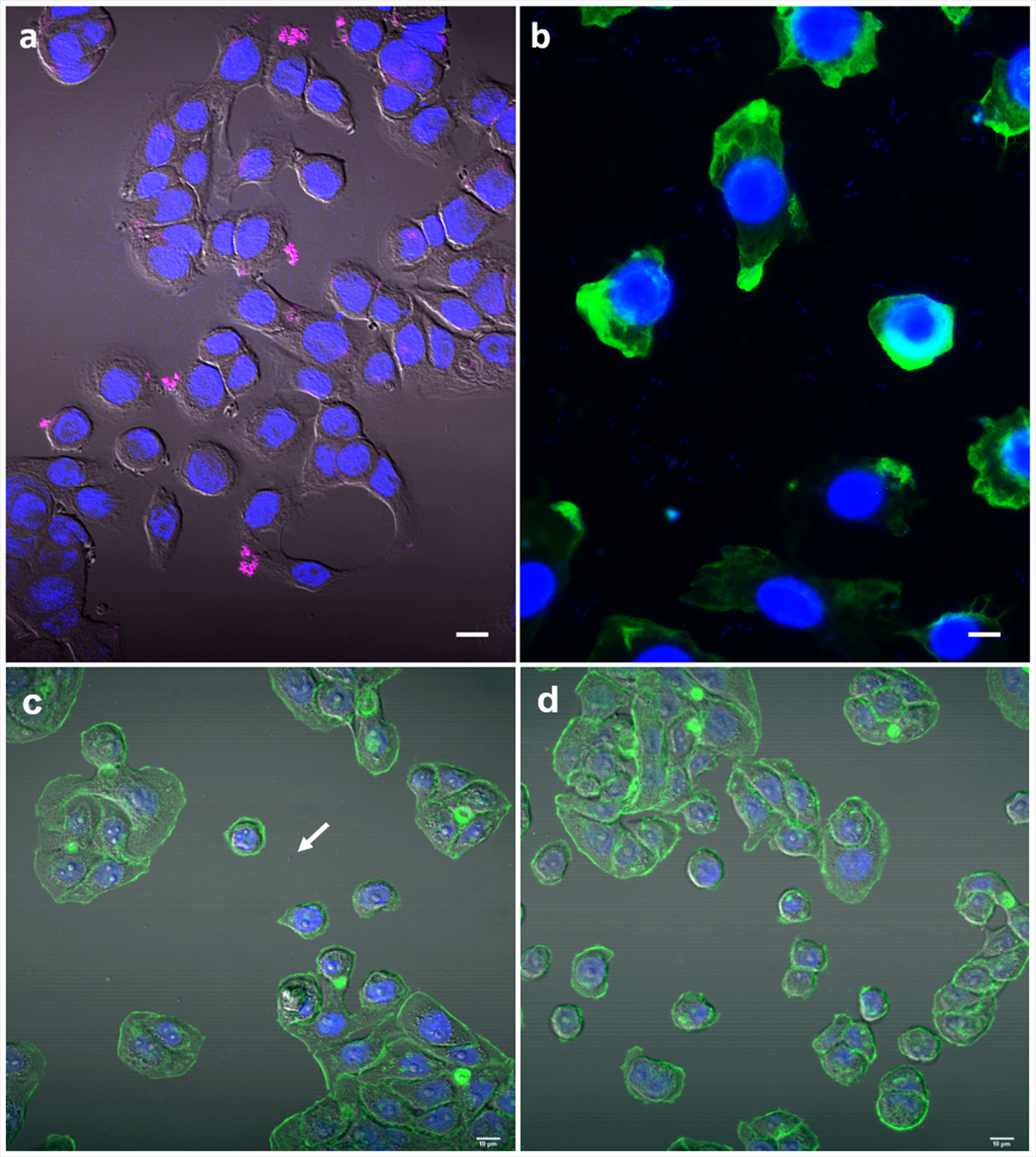

Supplement: Supplementary Figure 1 — C. striatum was used to determine specific adherence to HT-29 cells and as a control for localized adherence to HT-29 cells. Cells were infected for 3 h. C. striatum and the bacteria stained with a polyclonal antibody are shown in pink. The image in (b) was intentionally overexpose to show DAPI staining nuclei and bacteria. (c) Shows a non-adherent strain (neither to the cells nor the coverslips) and one control slide with cells alone (d). Arrow indicates a single bacterium to account for the assay. Magnification (a,c,d) ×400; (b) ×600. Scale bars indicate: (a,c,d) 10 μm; (b) 5 μm. [file Image_1.tif]

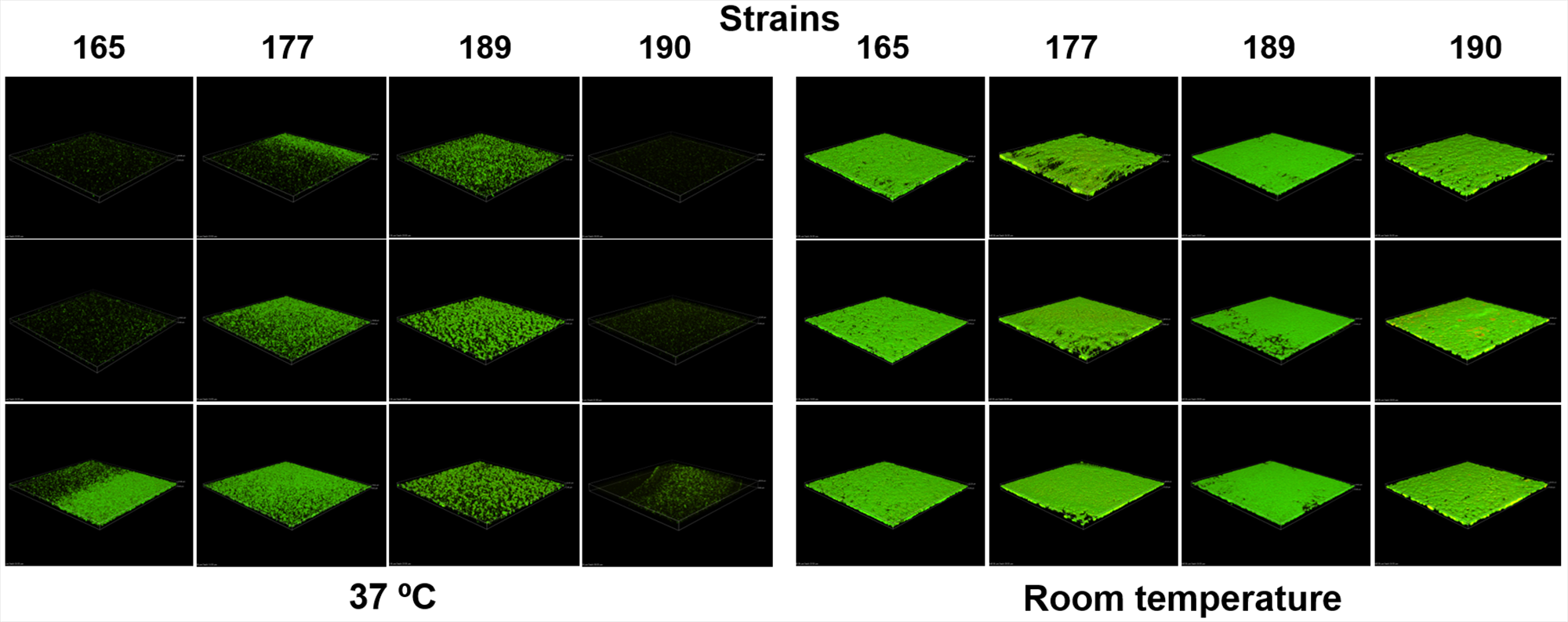

Supplement: Supplementary Figure 2 — CLSM of biofilm formation by selected Citrobacter strains at different temperatures. Representative examples of CLSM images of selected strains after biofilm formation. Bacteria were stained with the BacLight LIVE/DEAD viability kit. Live cells fluoresce in green with Syto 9 dye and dead cells are stained red with propidium iodide. Original magnification: ×200. [file Image_2.TIF]
