# Supplementary material for: Adherence to Human Colon Cells by Multidrug Resistant Enterobacterales Strains Isolated From Solid Organ Transplant Recipients With a Focus on Citrobacter freundii
Source: Front Cell Infect Microbiol. 2020 Sep 16;10:447. doi: 10.3389/fcimb.2020.00447 (PMC7525035; doi:10.3389/fcimb.2020.00447)
Supplement: Supplementary Table 1 — Strains that did not show adherence to colon cells. Strains that caused infection are highlighted in bold. [file Table_1.DOCX]

| ***E. coli*** | | | |
| --- | --- | --- | --- |
| **Strain** | **Nº weeks post-transplant*** | **Transplant** |  |
| 4 | 2 | Renal |  |
| 9 | 2 | Renal |  |
| 10 | 2 | Renal |  |
| 13 | 0** | Renal |  |
| 14 | 1 | Renal |  |
| 18 | 0** | Renal |  |
| 20 | 1 | Hepatic |  |
| 33 | 0** | Renal |  |
| 34 | 3 | Hepatic |  |
| 36 | 4 | Hepatic |  |
| 37 | 3 | Renal |  |
| 39 | 2 | Hepatic |  |
| 41 | 2 | Renal |  |
| 43 | 1 | Hepatic |  |
| 44 | 1 | Renal |  |
| 48 | 2 | Hepatic |  |
| 50 | 3 | Renal |  |
| 52 | 2 | Renal |  |
| 53 | 3 | Renal |  |
| 56 | 0** | Renal |  |
| 61 | 0** | Renal |  |
| 62 | 1 | Hepatic |  |
| 70 | 0** | Renal |  |
| 78 | 4 | Renal |  |
| 81 | 0** | Renal |  |
| 82 | 4 | Renal |  |
| 86 | 4 | Hepatic |  |
| 87 | 3 | Hepatic |  |
| 88 | 3 | Hepatic |  |
| 90 | 2 | Hepatic |  |
| 98 | 3 | Hepatic |  |
| 100 | 0** | Hepatic |  |
| 101 | 0** | Hepatic |  |
| 105 | 0** | Hepatic |  |
| 107 | 3 | Renal |  |
| 108 | 3 | Renal |  |
| 109 | 1 | Renal |  |
| 118 | 1 | Renal |  |
| 123 | 1 | Renal |  |
| 125 | 3 | Renal |  |
| 127 | 1 | Hepatic |  |
| 128 | 4 | Hepatic |  |
| 134 | 0** | Hepatic |  |
| 141 | 5 | Renal |  |
| 142 | 0** | Hepatic |  |
| 143 | 3 | Hepatic |  |
| 145 | 0** | Renal |  |
| 151 | 3 | Renal |  |
| 164 | 1 | Renal |  |
| 170 | 0** | Renal |  |
| 179 | 1 | Renal |  |
| 187 | 2 | Hepatic |  |
| 188 | 3 | Renal |  |
| 192 | 0** | Renal |  |
| 194 | 4 | Hepatic |  |
| 196 | 4 | Hepatic |  |
| 200 | 0** | Hepatic |  |
| 204 | 1 | Hepatic |  |
| 208 | 3 | Hepatic |  |
| 212 | 1 | Hepatic |  |
| 213 | 3 | Renal |  |
| 214 | 1 | Renal |  |
| 217 | 5 | Hepatic |  |
| 221 | 3 | Renal |  |
| 222 | 1 | Renal |  |
| 223 | 5 | Renal |  |
| 225 | 1 | Renal |  |
| 227 | 1 | Renal |  |
| 229 | 4 | Renal+pancreatic |  |
| 232 | 4 | Renal |  |
| 234 | 4 | Renal+pancreatic |  |
| 236 | 2 | Renal |  |
| 237 | 3 | Renal |  |
|  |  |  |  |
| ***K. pneumoniae*** | | |  |
| **Strain** | **Nº weeks post-transplant*** | **Transplant** |  |
| 2 | 2 | Renal |  |
| 3 | 2 | Renal |  |
| 8 | 2 | Renal |  |
| 12 | 3 | Renal |  |
| 15 | 3 | Renal |  |
| 16 | 2 | Hepatic |  |
| 17 | 5 | Hepatic |  |
| 19 | 3 | Renal |  |
| **27** | **Skin abscess** | **Renal** |  |
| 29 | 2 | Renal |  |
| 35 | 4 | Renal |  |
| 38 | 2 | Hepatic |  |
| 45 | 2 | Hepatic |  |
| 54 | 2 | Hepatic |  |
| 58 | 1 | Renal |  |
| 60 | 1 | Hepatic |  |
| 63 | 1 | Hepatic |  |
| 64 | 1 | Renal+pancreatic |  |
| 65 | 2 | Renal+pancreatic |  |
| 67 | 0** | Hepatic |  |
| 71 | 5 | Renal |  |
| 72 | 6 | Renal |  |
| 75 | 2 | Renal |  |
| 76 | 2 | Renal |  |
| 77 | 2 | Renal |  |
| 80 | 0** | Renal |  |
| 84 | 0** | Hepatic |  |
| 89 | 3 | Hepatic |  |
| 102 | 0** | Hepatic |  |
| 106 | 2 | Hepatic |  |
| 111 | 0** | Renal |  |
| 112 | 4 | Renal |  |
| 117 | 2 | Renal |  |
| 119 | 0** | Renal |  |
| 161 | 4 | Hepatic |  |
| **162** | **blood** | **Hepatic** |  |
| 168 | 4 | Hepatic |  |
| 169 | Bile | Hepatic |  |
| 171 | 1 | Renal |  |
| **172** | **Urine** | **Renal** |  |
| **173** | **Urine** | **Renal** |  |
| 174 | 5 | Renal |  |
| 175 | 6 | Renal |  |
| 176 | 1 | Renal |  |
| 180 | 3 | Hepatic |  |
| 182 | 5 | Hepatic |  |
| 191 | 1 | Hepatic |  |
| 193 | 3 | Hepatic |  |
| 197 | 3 | Renal |  |
| 203 | 1 | Hepatic |  |
| 205 | 1 | Hepatic |  |
| 210 | 1 | Hepatic |  |
| 215 | 4 | Renal |  |
| 216 | 1 | Hepatic |  |
| 220 | 5 | Hepatic |  |
| 238 | 1 | Hepatic |  |
| **239** | **Infection** | **Hepatic** |  |
|  |  |  |  |
| ***Enterobacter* spp.** | | | |
| **Strain** | **Nº weeks post-transplant*** | **Transplant** | **Species** |
| 1 | 3 | Renal | *E. cloacae* |
| 11 | 1 | Renal | *E. cloacae* |
| 24 | 3 | Renal | *E. cloacae* |
| 49 | 2 | Hepatic | *E. aerogenes* |
| 73 | 2 | Renal | *E. cloacae* |
| 130 | 1 | Renal | *E. cloacae* |
| 132 | 3 | Renal | *E. cloacae* |
| 133 | 4 | Renal | *E. cloacae* |
| 139 | 2 | Renal | *E. cloacae* |
| 140 | 3 | Renal | *E. cloacae* |
| 146 | 3 | Renal | *E. asburiae* |
| 148 | 0** | Renal | *E. cloacae* |
| 167 | 2 | Renal | *E. aerogenes* |
| 183 | 1 | Renal+pancreatic | *E. cloacae* |
| 206 | 1 | Hepatic | *E. cloacae* |
| 226 | 1 | Hepatic | *E. cloacae* |
| 233 | 4 | Renal | *E. aerogenes* |
| 240 | 0** | Hepatic | *E. cloacae* |
|  |  |  |  |
| ***Citrobacter* spp.** | | | |
| **Strain** | **Nº weeks post-transplant*** | **Transplant** | **Species** |
| 28 | 2 | Renal | *C. freundii* |
| 42 | 1 | Hepatic | *C. freundii* |
| 66 | 1 | Hepatic | *C. freundii* |
| 122 | 1 | Renal | *C. freundii* |
| 147 | 0** | Renal | *C. freundii* |
| 153 | 2 | Hepatic | *C. freundii* |
| 157 | 3 | Hepatic | *C. freundii* |
| 165 | 1 | Renal | *C. freundii* |
| 189 | 4 | Hepatic | *C. freundii* |
| 190 | 2 | Renal | *C. freundii* |
| 218 | 4 | Hepatic | *C. braakii* |
| 219 | 1 | Renal | *C. freundii* |
| 224 | 4 | Renal | *C. freundii* |
| 230 | 2 | Renal | *C. freundii* |
| 231 | 3 | Renal | *C. freundii* |
| 241 | 3 | Hepatic | *C. freundii* |
| 242 | 1 | Renal | *C. freundii* |
| 243 | 3 | Renal | *C. freundii* |
| 244 | 0** | Renal | *C. freundii* |

*Week in which the strain was isolated from rectal swab after transplantation.

** Strain isolated from rectal swab before transplantation.
